# Elicitation of Diosgenin Production in *Trigonella foenum-graecum* (Fenugreek) Seedlings by Methyl Jasmonate

**DOI:** 10.3390/ijms161226208

**Published:** 2015-12-15

**Authors:** Spandan Chaudhary, Surendra K. Chikara, Mahesh C. Sharma, Abhinav Chaudhary, Bakhtiyar Alam Syed, Pooja S. Chaudhary, Aditya Mehta, Maulik Patel, Arpita Ghosh, Marcello Iriti

**Affiliations:** 1 Department of Medical Genetics, Xcelris Labs Limited, Old Premchandnagar Road, Opp. Satyagrah Chhavani, Bodakdev, Ahmedabad-380015, Gujarat, India; spandan.chaudhary@gmail.com (S.C.); surendrakchikara@gmail.com (S.K.C.); abhinavchaudhary26@yahoo.co.in (A.C.); pooja.chaudhary@xcelrislabs.com (P.S.C.); aditya.mehta@xcelrislabs.com (A.M.); maulik.patel@xcelrislabs.com (M.P.); arpita.ghosh@xcelrislabs.com (A.G.); 2 Department of Biotechnology, Kadi Sarva Vishwavidyalaya, II Floor, KBIPER Building, Kadi Campus, Sector-23, Gandhinagar-382023, Gujarat, India; mc_sharma2@rediffmail.com; 3 Department of Biotechnology, Hemchandracharya North Gujarat University, Patan-384265, Gujarat, India; samisyed40@gmail.com; 4 Department of Agricultural and Environmental Sciences, Milan State University, via G. Celoria 2, Milan 20133, Italy

**Keywords:** elicitors, mevalonate pathway, steroidal saponins, 3-hydroxy-3-methylglutaryl-CoA reductase, sterol-3-β-glucosyl transferase, plant innate immunity

## Abstract

The effects of methyl jasmonate (MeJA), an elicitor of plant defense mechanisms, on the biosynthesis of diosgenin, a steroidal saponin, were investigated in six fenugreek (*Trigonella foenum-graecum*) varieties (Gujarat Methi-2, Kasuri-1, Kasuri-2, Pusa Early Branching, Rajasthan Methi and Maharashtra Methi-5). Treatment with 0.01% MeJA increased diosgenin levels, in 12 days old seedlings, from 0.5%–0.9% to 1.1%–1.8%. In addition, MeJA upregulated the expression of two pivotal genes of the mevalonate pathway, the metabolic route leading to diosgenin: 3-hydroxy-3-methylglutaryl-CoA reductase (*HMG*) and sterol-3-β-glucosyl transferase (*STRL*). In particular, MeJA increased the expression of *HMG* and *STRL* genes by 3.2- and 22.2-fold, respectively, in the Gujarat Methi-2 variety, and by 25.4- and 28.4-fold, respectively, in the Kasuri-2 variety. Therefore, MeJA may be considered a promising elicitor for diosgenin production by fenugreek plants.

## 1. Introduction

*Trigonella foenum-graecum* (fenugreek) is a dicotyledonous plant belonging to the family of Fabaceae (subfamily Papilionaceae), widely used for a number of medicinal applications all over the world [1]. Fenugreek has been extensively used, in Indian Ayurveda and Chinese Traditional Medicine, as remedy for treatment of illnesses and conditions including epilepsy, paralysis, gout, dropsy, chronic cough, diabetes, piles, sinus, lung congestion, inflammation, infections, as well as in hair treatment, breast enhancement and for its aphrodisiac effects [2,3,4]. In addition, fenugreek plants possess anticancer, anti-fertility, anti-ageing, antimicrobial, anti-parasitic, galactagogue and hypocholesterolaemic effects [5,6,7,8,9,10,11,12,13,14,15]. Many bioactive phytochemicals have been reported in fenugreek, including saponins, a class of glycosylated triterpenes that show antimicrobial, antiviral and insecticidal activities [16]. These compounds represent a part of the plant defense mechanisms against biotic stress and, therefore, can also be classified as phytoprotectants [17,18]. Among steroidal saponins, diosgenin ((25*R*)-5-spirosten-3H-ol) (Pubchem ID 99474) is one of the most bioactive components of fenugreek which has been investigated due to its anticancer and anti-aging activities [19,20,21,22,23]. It is also used as contraceptive and to prevent cardiovascular diseases [24,25,26,27,28]. Notably, the synthesis of some steroidal drugs and hormones such as testosterone, glucocorticoids, norethisterone and progesterone may arise from diosgenin used as precursor [9].

Currently, certain wild species of Mexican yam (*Dioscorea* spp.) are used for diosgenin production, even if they need a long time to accumulate high diosgenin levels [29]. Therefore, fenugreek may represent a more suitable system for diosgenin production because of short growth cycle and low cost of production [30,31]. The maximum amount of diosgenin naturally present in fenugreek is found in young leaves and in mature seeds, with percentages ranging from 0.28% to 0.92%. However, to develop fenugreek as a source of diosgenin, it is pivotal to trigger the cascade of 11 key genes responsible for diosgenin biosynthesis identified in our previous study [32].

The aim of the present study is to increase the content of diosgenin in fenugreek by treatment with methyl jasmonate (MeJA), an elicitor able to trigger the plant defense mechanisms. MeJA was first isolated from the plant pathogenic fungus *Lasiodiplodia theobromae* as plant growth inhibitor and the role of jasmonic acid, in elicitor-induced signal transduction pathway, was first described by Gundlach *et al.* [33,34]. Then, MeJA, a plant growth regulator, was reported to increase the accumulation of secondary metabolites involved in plant resistance against pathogens [1,35,36,37,38,39,40]. It triggers a cascade of intracellular signals and also activates *de novo* transcription of genes as phenylalanine ammonia-lyase (PAL), the key enzyme of the phenylpropanoid pathway leading to accumulation of antimicrobial phytoalexins [41]. It is also known that the 3-hydroxy-3-methylglutaryl-CoA reductase (HMGR) is an important rate limiting and key regulatory enzyme for isoprenoid or mevalonate biosynthetic route in plants, that catalyzes the irreversible conversion of 3-hydroxy-3-methylglutaryl-CoA (HMG) to mevalonate. Therefore, expression of this enzyme increases the total sterol and cycloartenol accumulations by two and 100-fold, respectively [42]. Two pivotal genes of the metabolic pathway leading to diosgenin, 3-hydroxy-3-methylglutaryl-CoA reductase (*HMG*) and sterol-3-β-glucosyl transferase (*STRL*), were selected as target genes for the current study, in order to investigate their involvement in diosgenin biosythesis.

## 2. Results

**Figure 1:**
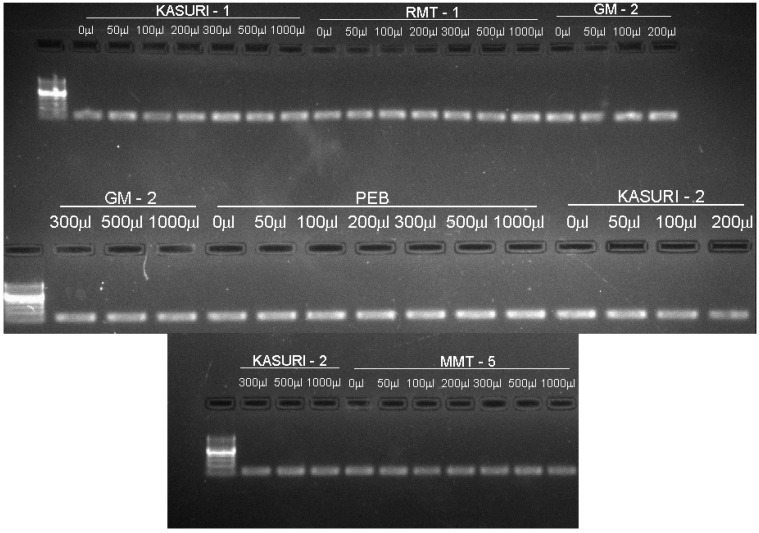
Electrophoretogram of amplified products generated for first strand complementary DNA (cDNA) from total RNA of methyl jasmonate (MeJA)-treated and control fenugreek (*Trigonella foeum-graecum* L.) plants using 18 housekeeping gene primers, analysed using 2% agarose gel electrophoresis.

Total RNAs extracted from 36 treated and six control samples, analyzed with two peaks representing 28S and 18S ribosomal RNA along with a 5S ribosomal RNA peak, were observed on denaturing agarose gel electrophoresis and the RNA integrity number (RIN) value of all of them was found to be above six, which indicates that RNA can be used for further analyses. The size of the amplicons generated using housekeeping gene primers was 180 bp as confirmed in the agarose gel electrophoresis of all the 42 samples (Figure 1; Table 1). The amplicons generated for Kasuri-I and Kasuri-II variety were of low intensity, indicative of variation in the GC (guanine-cytosine) content of this plant.

**Figure 2:**
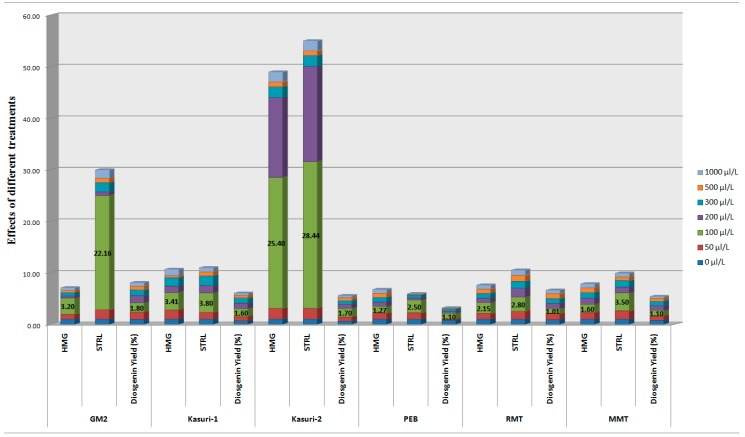
Effects of methyl jasmonate (MeJA) treatments on the expression of 3-hydroxy-3-methylglutaryl-CoA reductase (*HMG*) and sterol-3-β-glucosyl transferase (*STRL*) genes and diosgenin yield (%) in fenugreek (*Trigonella foeum-graecum* L.) plants (as per data reported in Supplementary Table S1).

The expression of 3-hydroxy-3-methylglutaryl-CoA reductase (*HMG*) and sterol-3-β-glucosyl transferase (*STRL*) genes and diosgenin content of fenugreek seedlings are reported in Figure 2. Pattern of expression of both genes was found to be different among the fenugreek varieties, even if the highest expression was observed at 100 µL/L (0.01%) MeJA, in all varieties. Maximum expression of *HMG* gene was 3.2-fold in GM-2, 3.41-fold in Kasuri-1, 25.4-fold in Kasuri-2, 1.27-fold in PEB, 2.15 folds in RMT and 1.6 folds in MMT seedlings treated with 100 µL/L MeJA. The maximum expression of *STRL* gene was 22.16, 3.8, 28.44, 2.5, 2.8, and 3.5 folds in GM-2, Kasuri-1, Kasuri-2, PEB, RMT and MMT seedlings, respectively, after treatment with 100 µL/L MeJA. A two to three fold increase of diosgenin was recorded in all fenugreek varieties treated with 100 µL/L MeJA, in accordance with gene expression results (Figure 2). Notably, the eliciting effect of MeJA was shown to be dose-dependent on the expression of both target genes as well as on diosgenin levels, decreasing with increase of MeJA concentration up to 1000 µL/L (Table 2). Both results on gene expression and diosgenin content were statistically significant (*p* < 0.05) for 100 µL/L MeJA. However, the effects of MeJA treatments on seedling length, fresh weight and dry weight were not statistically significant (*p* > 0.05) (Table 3, Table 4 and Table 5).

**Table 1:** Primer details of 3-hydroxy-3-methylglutaryl-CoA reductase (*HMG*), sterol-3-β-glucosyl transferase (*STRL*) and 18S housekeeping genes.

Primer	Primer Sequence	Reference	PE ± SE *
18S Ribosomal RNA	F primer: 5′→3′ GTCTCAACCATAAACGATGCCGACCA	Accession ID: KJ813746	2.0 ± 0.0
	R primer: ACCTGGTAAGTTTCCCCGTGTTGAGT		
*HMG*	R primer: 5′→3′ ACTCAACACGGGGAAACTTACCAGGT	171 bp Contig 19639-our transcriptome data	2.1 ± 0.0
	F primer: 5′→3′ CACCCGCAAGATGAGATTTT		
*STRL*	F primer: 5′→3′ TGGAAAATGAGGATGGGGTA	144 bp Conting 10332-our transcriptome data	2.0 ± 0.0
	R primer: 3′→5′TTGCATTGGATCAGGAACAA		

* PE ± SE: primer efficiency ± standard error.

**Table 2:** Effect of methyl jasmonate (MeJA) treatments on diosgenin content (%) in fenugreek (*Trigonella foeum-graecum* L.) varieties.

MeJA Concentration	Fenugreek Varieties (Diosgenin Yield in %)					
	GM2	Kasuri-1	Kasuri-2	PEB	RMT	MMT
0 µL/L	0.9	0.65	0.55	0.75	0.85	0.75
50 µL/L	1.44	0.78	0.83	0.23	1.19	0.83
100 µL/L	1.8 *	1.6 *	1.7 *	1.1 *	1.01 *	1.1 *
200 µL/L	0.72	1.04	0.77	0.38	0.97	0.9
300 µL/L	1.62	1.01	0.66	0.45	1.02	0.83
500 µL/L	0.72	0.48	0.61	0.15	0.94	0.6
1000 µL/L	1.36	0.46	0.44	0.11	0.68	0.38

* *p* < 0.05 significant by ANOVA with *post-hoc* Tukey HSD test.

**Table 3:** Effect of methyl jasmonate (MeJA) treatments on the length (cm) of seedlings of different fenugreek (*Trigonella foeum-graecum* L.) varieties.

Fenugreek Varieties	MeJA Treatment						
	0 µL/L	50 µL/L	100 µL/L	200 µL/L	300 µL/L	500 µL/L	1000 µL/L
GM-2	5.5 ± 2 *	5.5 ± 3	5.2 ± 4	5.5 ± 3	5.8 ± 4	5.9 ± 2	5.8 ± 4
K-1	4.8 ± 3	4.9 ± 2	4.3 ± 4	5.2 ± 2	5.1 ± 3	5 ± 2	4.8 ± 3
K-2	4.6 ± 2	4.8 ± 3	4.2 ± 3	5 ± 2	4.9 ± 3	4.8 ± 2	4.9 ± 2
PEB	5.2 ± 3	5.2 ± 2	4.3 ± 3	4.9 ± 2	4.8 ± 2	4.9 ± 3	4.8 ± 2
RMT	7.2 ± 4	7.2 ± 3	6.4 ± 2	7 ± 3	9.8 ± 5	6.9 ± 3	7 ± 3
MMT	6.2 ± 3	6.2 ± 2	5.3± 4	6.2 ± 3	6 ± 2	5.9 ± 3	5.8 ± 4

* Mean value ± SD (*n* = 3); here *p* > 0.05 indicates data not significant.

**Table 4:** Effect of methyl jasmonate (MeJA) treatments on the fresh weight (mg) of seedlings of different fenugreek (*Trigonella foeum-graecum* L.) varieties.

Fenugreek Varieties	MeJA Treatment						
	0 µL/L	50 µL/L	100 µL/L	200 µL/L	300 µL/L	500 µL/L	1000 µL/L
GM-2	175 ± 1 *	172 ± 4	180 ± 8	179 ± 0	178 ± 5	174 ± 6	173 ± 5
K-1	145 ± 3	151 ± 2	152 ± 1	146 ± 5	147 ± 0	142 ± 2	140 ± 2
K-2	140 ± 5	144 ± 8	148 ± 8	142 ± 7	142 ± 5	141 ± 2	140 ± 9
PEB	179 ± 5	175 ± 1	182 ± 3	173 ± 8	172 ± 8	170 ± 5	168 ± 5
RMT	190 ± 8	185 ± 2	192 ± 5	187 ± 0	184 ± 8	183 ± 5	180 ± 3
MMT	174 ± 6	176 ± 1	180 ± 7	178 ± 2	177 ± 5	175 ± 6	171 ± 3

* Mean value ± SD (*n* = 3); here *p* > 0.05 indicates data not significant.

**Table 5:** Effect of methyl jasmonate (MeJA) treatments on the dry weight (mg) of seedlings of different fenugreek (*Trigonella foeum-graecum* L.) varieties.

Fenugreek Varieties	MeJA Treatment						
	0 µL/L	50 µL/L	100 µL/L	200 µL/L	300 µL/L	500 µL/L	1000 µL/L
GM-2	130 ± 8 *	128 ±1	134 ±9	131 ± 5	124 ± 5	122 ±5	120 ± 8
K-1	85 ± 5	88 ± 3	92 ± 5	81 ± 5	83 ± 0	82 ± 8	79 ± 8
K-2	80 ± 5	82 ± 5	85 ± 9	84 ± 8	83 ± 7	79 ± 8	78 ± 3
PEB	125 ± 5	124 ± 9	136 ± 8	134 ± 0	133 ± 8	128 ± 8	122 ± 2
RMT	135 ± 5	136 ± 0	142 ± 5	139 ± 8	138 ± 5	132 ± 7	130 ± 5
MMT	133 ± 9	134 ± 8	140 ± 5	138 ± 7	137 ± 1	136 ± 5	130 ± 2

* Mean value ± SD (*n* = 3); here *p* > 0.05 indicates data not significant.

## 3. Discussion

The occurrence of diosgenin in plants is usually very low, even if its levels may be enhanced by treatment with elicitors or under stress conditions. A number of studies have provided evidence that some plant growth regulators, as ethylene and ethephon (an ethylene donor), may elicit the diosgenin accumulation in plants. Diosgenin content in fenugreek increased by 195% and 126% after treatment with 25 and 5 ppm ethephon, respectively [43,44]. MeJA was previously shown to be the most effective elicitor on diosgenin concentration in fenugreek seedlings, with a 10.5-fold increase at 100 µL/L MeJA, though varietal differences and gene expression were not investigated in this study [45]. In general, increase of secondary metabolite production can be mediated by the expression of key genes involved in specific metabolic routes [46,47].

Plants are sessile organisms continuously exposed to a plethora of biotic and abiotic stresses, and secondary metabolites are produced as part of the plant defense mechanisms [48]. A number of studies have proved that MeJA is a powerful inducer of secondary metabolites in various plants [49,50,51]. It enhanced the cell biomass and induced the phytochemical production in some plants of the Lamiaceae family, with positive or neutral effects on plant growth [52]. Earlier studies also reported that MeJA, originally isolated from fungal cell wall, up-regulates the expression of *HMG* and other genes involved in mevalonate pathway in some fungal species [53].

**Figure 3:**
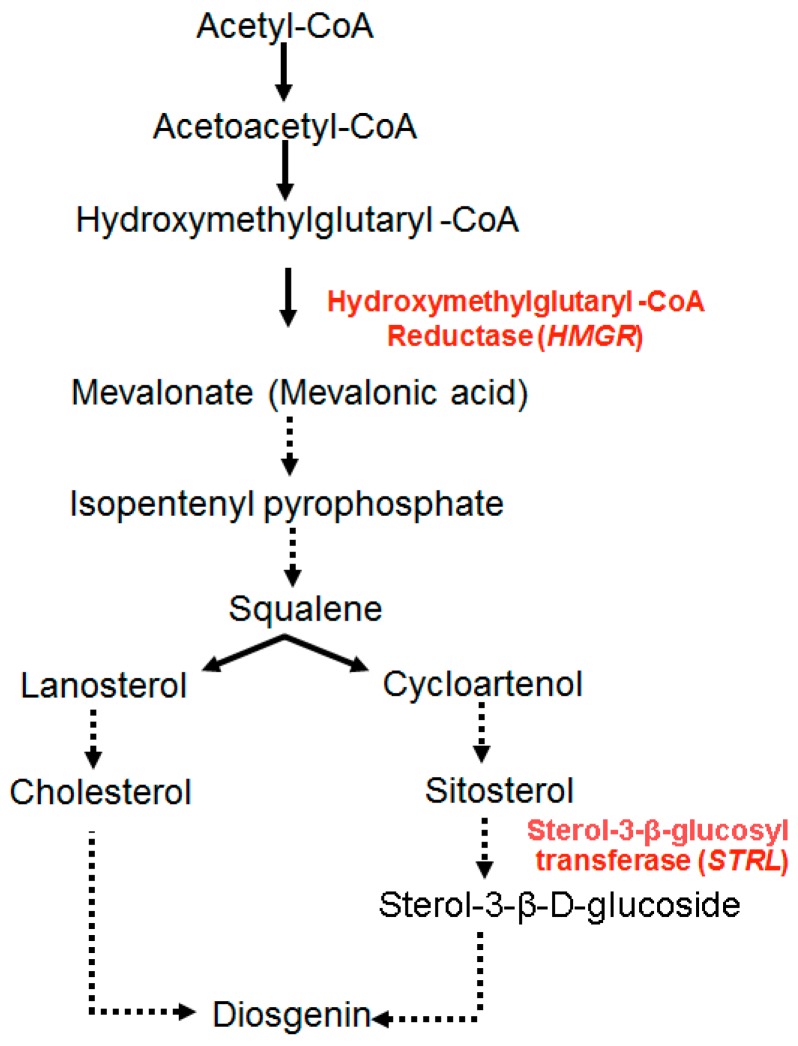
Proposed diosgenin biosynthetic pathway. 3-Hydroxy-3-methylglutaryl-CoA is converted to mevalonate by 3-hydroxy-3-methylglutaryl-CoA reductase (HMGR) enzyme. This is the major rate limiting step of isoprenoid pathway. Mevalonate is converted to isopentenyl pyrophosphate via series of multiple reactions. Then, diosgenin is produced from squalene by two routes: via cholesterol from lanosterol and via sitosterol from cycloartenol to sterol 3-β-d-glucoside. Finally, the latter is converted to diosgenin by sterol 3-β-glucosyl transferase (STRL) enzyme (dashed lines indicate multiple steps involved in the pathway; arrows indicate the flow of reaction in pathway).

The expression of *HMG* gene is central to diosgenin biosynthesis, as the HMGR enzyme regulates the rate limiting step of the isoprenoid pathway and provides mevalonate, the precursor for the production of both primary and secondary metabolites (Figure 3). Ethylene treatment enhanced both *HMG* gene expression and diosgenin levels in *in vitro* cultures of *Dioscorea zingiberensis* [54]. Similarly, MeJA treatment stimulated *HMG* gene in *Camptotheca acuminata* [55].

Based on previous fenugreek transcriptome studies [33], we proposed a pathway for the production of diosgenin by combining mevalonate and sterol metabolic routes (Figure 3). We hypothesised that diosgenin may be formed from squalene 2,3-oxide in two ways, (i) from lanosterol, via the formation of cholesterol; and (ii) from cycloartenol via sitosterol. Mehrafarin *et al.* [56] tried to explain the biosynthesis of steroidal saponins from cholesterol, though all the steps were not completely defined. In the present study, the expression of the second target gene, *STRL*, was also elicited by MeJA treatment more than two fold in comparison with the *HMG* gene, thus supporting the proposed hypothesis of diosgenin biosynthesis from squalene via cycloartenol and sitosterol. The effects of MeJA on the increased expression of the two target genes of mevalonate pathway can be explained on the basis of transcriptional modulation of related genes as shown to be a common response to elicitor signals [57].

## 4. Materials and Methods

### 4.1. Fenugreek Seed Sterilization and Sowing

Seeds of six fenugreek (*Trigonella foenum-graecum* L.) varieties Gujarat Methi-2 (GM-2), Kasuri-1, Kasuri-2, Pusa Early Branching (PEB), Rajasthan Methi (RMT-1) and Maharashtra Methi-5 (MMT-5) were sterilized using sodium hypochlorite solution (4% *W*/*V* Merck, UN1791) for 10 min. Sterilized seeds were soaked in autoclaved water in petri-dishes for 48 h in dark for sprouting process. The germinated seeds were transferred to the pots filled with autoclaved soil from a farm near Ahmedabad (latitude 23°03′N and longitude 72°40′E). Then, in each plastic container (3.5 cm diameter at the bottom and 5.5 cm diameter at the top with 5.5 cm of depth), approximately 50 g of soil were transferred and 30 sprouted seeds transplanted; these were grown for 6 days in a sterile room with a light:dark cycle of 10:14 h at 25 °C temperature with the relative humidity around 36%–40% without any treatment.

### 4.2. Methyl Jasmonate Treatments

Methyl jasmonate (MeJA, Sigma Aldrich, St. Louis, MO, USA, reference number 392707, 95% pure) was dissolved in absolute ethanol and diluted in water to obtain 50, 100, 200, 300, 500 and 1000 µL/L (0.005%, 0.01%, 0.02%, 0.03%, 0.05% and 0.1%) solutions. Ethanol was used as control. One mL of each dilution was administered to each pot, in the morning and evening, 7 and 9 days after sprouted seed transplantation and pots were watered with 20 mL of water for uniform drenching of pot soil. A total of 36 experiments (6 fenugreek varieties 6× MeJA concentrations) and 6 control pots were grown in triplicate. Some seedlings harvested on the 12th day were washed thoroughly with autoclaved water to remove soil from the roots and stored in TMS RNA stabilizer (XGtms-100) solution at −80 °C for RNA isolation and gene expression studies, while others were kept 5 days in a hot air oven at 50 °C till complete dryness for diosgenin extraction.

### 4.3. Growth Parameter Measurement

Length and fresh weight of seedlings were recorded before diosgenin extraction; then they were dried for 5 days in a hot air oven at 50 °C to complete dryness and dry weight was registered.

### 4.4. Diosgenin Extraction and Analysis

Diosgenin extraction and determination were carried out according to Trivedi *et al.* [58] with modifications. Briefly, dried fenugreek seedlings were ground to fine powder using liquid nitrogen and re-fluxed with 50 mL of 2.5 M ethanol-sulfuric acid at 80 °C for 4 h. The solution was then filtered using Whatman filter paper no.1, diluted with 50 mL double distilled water and extracted with 50 mL of *n*-hexane 3 times, pooled and evaporated to dryness at room temperature. Dry residues were dissolved in 25 mL mobile phase (acetonitrile:water 90:10) and filtered through 0.22 µm filter before high performance liquid chromatography (HPLC) analysis. For HPLC analysis, Hypersil ODS C18, 5 µm, 250 × 4.6 mm (Thermo Scientific, Waltham, MA, USA) was used at flow rate of 1 mL per minute with mobile phase (acetonitrile:water 90:10), at 35 °C for 30 min. One mg per mL of diosgenin (D1634, SIGMA, Sigma Aldrich, St. Louis, MO, USA) was used as standard [58].

### 4.5. Primer Designing

Primers for the two target genes 3-hydroxy-3-methylglutaryl-CoA reductase (*HMG*) and sterol-3-β-glucosyl transferase (*STRL*) were designed as in the whole transcriptome data of Gujarat Methi-1 variety published by our lab [55]. Primer 3 plus software was used for primer designing and designed primers were synthesized in Primex facility at our lab. Primer efficiency of genes and their relative expressions were calculated using relative expression software tool [59]. Primer details are presented in Table 1.

### 4.6. RNA Extraction, Complementary DNA (cDNA) Preparation and Gene Expression

Fifty mg of frozen seedlings from all 42 pots were used for RNA isolation using Xcelgen plant RNA miniprep kit (XG6611-01) following the manufacturer’s instructions. The quantity of isolated total RNA was determined by absorbance ratios A260/280 and A260/230, on nanodrop spectrophotometer 8000 (Thermo Scientific), and quality of total RNA was analysed, on 1% denaturing agarose gel and Agilent Bioanalyzer 2100, using the RNA 6000 pico chip (Agilent Technologies, Santa Clara, Palo Alto, CA, USA). Then, 500 ng of total RNA was subjected to reverse transcription reaction for first strand cDNA synthesis using M-MLV reverse transcriptase enzyme (XG02032). For preparing first strand cDNA, 500 ng of total RNA with 0.5 µL RNase inhibitor were used with oligo dT primers. Reaction mixture used for cDNA synthesis contained 500–1000 ng of total RNA, 10 mM oligo-dt primer, 0.5 µL RNAse inhibitor and 10 mM dNTPs and the reaction was incubated at 65 °C for RNA denaturation. After incubation, 200 units of M-MLV enzyme, reverse trancriptase buffer and 50 mM dithiothreitol were added to the reaction and subjected to reverse transcription using three temperatures 25 °C for 10 min, 40 °C for 60 min and 70 °C for 10 min. First strand cDNA was quantified using nanodrop spectrophotometer and 100 ng were used for gene expression reaction. For standardization, polymerase chain reaction was set up in a gradient thermal cycler (Applied Biosystems Verity 96 well thermal cycler, 0.2 mL, Foster City, CA, USA) followed by analysis of the amplicons on 2% agarose gel. After standardization of the PCR conditions, the same conditions were used for gene expression assay using Light cycler 480II instrument (Roche lifesciences, Indianapolis, IN, USA). The gene expression reaction mixture contained 1X SYBR green master mix (Light cycler 480 SYBR green I master, 04707516001), 0.5 pM of each forward and reverse primers, 100 ng of first strand cDNA and 3 µL of nuclease free water (total volume 10 μL). All the samples were amplified using SYBR chemistry in triplicates using light cycler 480II instrument and CP values (crossing point (PCR-cycle) value) were recorded the same as *C*_t_ value (cycle threshold value); calculation of ΔΔ*C*_t_ was carried out using average *C*_t_ value and to analyze the gene expression pattern. Basic relative quantification method was used to analyze results as for studying physiological changes in the transcript level, as relative expression study is the most useful method [60]. Final results of the ΔΔ*C*_t_ value were described as fold change in the gene expression value.

### 4.7. Statistical Analysis

Statistical differences between control and treatments were assessed by one-way analysis of variance (ANOVA) with *post-hoc* Tukey HSD test. The *p*-value < 0.05 was considered significant.

## 5. Conclusions

In this study, our results showed that the 100 µL/L (0.01%) MeJA treatment increased diosgenin levels in seedlings of different fenugreek varieties, from 0.5%–0.9% (naturally occurring) to 1.1%–1.8%. In open-field conditions, it would require 1.24 µL/L MeJA which amounts, approximately, to $100/Ha crop area. The returns from increased diosgenin production might amount to around $100,000/Ha. MeJA elicited the expression of *HMG* and STRL genes, thus upregulating the mevalonate and sterol biosynthetic pathways and increasing diosgenin production from cycloartenol via the intermediate sitosterol. Therefore, MeJA may be considered a promising elicitor for diosgenin production by fenugreek plants.

## Supplementary Materials

Supplementary materials can be found at http://www.mdpi.com/1422-0067/16/12/26208/s1.
