# Supplementary material for: Elicitation of Diosgenin Production in Trigonella foenum-graecum (Fenugreek) Seedlings by Methyl Jasmonate
Source: Int J Mol Sci. 2015 Dec 15;16(12):29889–99. doi: 10.3390/ijms161226208 (PMC4691151; doi:10.3390/ijms161226208)
Supplement: Supplementary file 1 [file ijms-16-26208-s001.pdf]

Supplementary Materials: Elicitation of Diosgenin Production in *Trigonella foenum-graecum* (Fenugreek) Seedlings by Methyl Jasmonate

Spandan Chaudhary, Surendra K. Chikara, Mahesh C. Sharma, Abhinav Chaudhary, Bakhtiyar Alam Syed, Pooja S. Chaudhary, Aditya Mehta, Maulik Patel, Arpita Ghosh and Marcello Iriti

Table S1. Effects of methyl jasmonate (MeJA) treatments on the expression of 3-hydroxy-3-methylglutaryl-CoA reductase (*HMG*) and sterol-3-β-glucosyl transferase (*STRL*) genes and diosgenin yield (%) in fenugreek (*Trigonella foeum-graecum* L.) plants.

Fenugreek Variety	Metabolite Yield	Concentration of MeJA						
		00 µL/L	50 µL/L	100 µL/L	200 µL/L	300 µL/L	500 µL/L	1000 µL/L
GM2	HMG (FC)	1.00	0.88	3.20	0.38	0.60	0.44	0.56
	STRL (FC)	1.00	1.80	22.16	0.66	1.82	0.83	1.66
	Diosgenin %	0.90	1.44	1.80	0.72	1.62	0.72	1.36
Kasuri-1	HMG (FC)	1.00	1,77	3.41	1.19	1.62	0.40	1.25
	STRL (FC)	1.00	1.27	3.80	1.39	1.89	0.75	0.84
	Diosgenin %	0.65	0.78	1.60	1.04	1.01	0.48	0.46
Kasuri-2	HMG (FC)	1.00	2.08	25.40	15.50	2.11	0.91	1.95
	STRL (FC)	1.00	2.08	28.44	18.50	2.11	0.91	1.95
	Diosgenin %	0.55	0.83	1.70	0.77	0.66	0.61	0.44
PEB	HMG (FC)	1.00	1.15	1.27	0.87	0.85	0.83	0.76
	STRL (FC)	1.00	1.20	2.50	0.38	0.57	0.14	0.13
	Diosgenin %	0.75	0.23	1.10	0.38	0.45	0.15	0.11
RMT	HMG (FC)	1.00	1.07	2.15	0.81	0.90	0.85	0.80
	STRL (FC)	1.00	1.48	2.80	1.71	1.31	1.17	1.02
	Diosgenin %	0.85	1.19	1.01	0.97	1.02	0.94	0.68
MMT	HMG (FC)	1.00	1.23	1.60	1.20	1.10	0.90	0.80
	STRL (FC)	1.00	1.58	3.50	1.08	1.30	0.67	0.77
	Diosgenin %	0.75	0.83	1.10	0.90	0.83	0.60	0.38

(FC = fold change).
